# Interaction Mechanism between Slags and Alkali Silicate Activators: An Approach Based on the Al Phases

**DOI:** 10.3390/ma16217032

**Published:** 2023-11-03

**Authors:** Yu Jin, Weipeng Feng, Dapeng Zheng, Zhijun Dong

**Affiliations:** 1 Institute of Technology for Marine Civil Engineering, Shenzhen Institute of Information Technology, Shenzhen 518172, China; jinyu@sziit.edu.cn (Y.J.); fengwp@sziit.edu.cn (W.F.); 2 Key Laboratory for Resilient Infrastructures of Coastal Cities (MOE), College of Civil and Transportation Engineering, Shenzhen University, Shenzhen 518060, China; dapengzheng@szu.edu.cn

**Keywords:** alkali-activated slag, water glass, dissolution–reprecipitation mechanism, non-bridging oxygen model

## Abstract

In this study, we examined the early-stage interaction of three types of slag and six activators with different chemical compositions. To determine the degree of hydration (DOH) and hydrate assemblage in alkali-activated slag (AAS), we employed EDX, XRD, and NMR analyses. We found that with increasing silicate concentration in the activator, the DOH in the AAS varied, whereas the proportion of C-(N)-A-S-H increased and the other Al-containing phase decreased. When examining the impact of the activator on glass dissolution, it is apparent that an index based on the degree of depolymerization of the glass structure correlates with the DOH and the proportion of hydrotalcite in the AAS. Coupled with the activator’s modulus, this index can be utilised to elucidate the dissolution–reprecipitation mechanism that governs the interaction between the activator and slag.

## 1. Introduction

Sodium hydroxide and sodium silicate activated blast furnace slags are the most mature alkali activated materials available to date [1,2]. In order to utilize their potential within industry, it is imperative to develop a stable binder from a specific precursor under optimized activation conditions. Conversely, even a minor chemical variation between slags can have a substantial impact on the reaction kinetics of alkali-activated slags [3]. A thorough comprehension of the interaction between slag and activator is necessary to establish a scientific foundation for the mix design of alkali-activated slag (AAS) binders by refining the activation conditions.

The significance of slag chemistry variability regarding the AAS reaction has been widely acknowledged by researchers for many years [1]. Recently, researchers have conducted some systematic studies to establish a clear correlation between slag chemistry and hydrate assemblage in AAS. Ben Haha et al. [4] examined slags with different MgO content (8–13 wt.%) that were activated with both sodium hydroxide and sodium metasilicate (Na_2_SiO_3_·5H_2_O). The results illustrated that increasing the MgO content results in a faster reaction and greater hydrotalcite quantities. Meanwhile, the authors found that the calcium (sodium) aluminosilicate hydrate (C-(N)-A-S-H) gel had a reduced Al incorporation when activated by sodium metasilicate alone. Additionally, they conducted further studies on slags with varying Al_2_O_3_ content (8–17 wt.%), which showed that the extent of the early-stage reaction decreases as the Al_2_O_3_ content increases [5]. Consequently, the Mg-Al ratio in hydrotalcite decreases, the Al incorporation in C-(N)-A-S-H increases, and the formation of strätlingite occurs. The impact of Al_2_O_3_ content on the degree of reaction has been found to rely on the activator employed [6]. Similarly, the effect of CaO/SiO_2_ on the reaction of AAS is also dependent on the activator in use. When producing AAS with slag containing less than 5 wt.% MgO content, zeolitic products such as gismondine (CaAl_2_Si_2_O_8_·4H_2_O), garronite-Ca (Na_2_Ca_5_Al_12_Si_20_O_64_·27H_2_O) [7], and monocarboaluminate (4CaO·Al_2_O_3_·CO_2_·11H_2_O) [8] are formed. Furthermore, it has been reported that a third aluminate hydrate (TAH) forms in AAS [9]. TAH is a nanoscale aluminate hydrate with sixfold coordination associated with C-S-H gels [9,10]. However, some argue that the origin of the observed sixfold coordinated Al in the ^27^Al NMR spectrum is [AlO_2_(OH)_4_]^5−^ at the bridging site of the C-A-S-H gel [11].

The impact of activator type and dosage on the hydration products of AAS is a crucial concern of the research community [12,13,14,15,16,17]. AAS activated with sodium hydroxide or sodium silicate (water glass) yield the primary hydration products of CaO-(Na_2_O)-Al_2_O_3_-SiO_2_-H_2_O [C-(N)-A-S-H] and hydrotalcite [14,15,18,19,20,21,22,23]. At an equal level of alkali dosage, C-(N)-A-S-H produced in sodium hydroxide-activated slag generally exhibits a lower level of cross-linking than that in sodium silicate-activated slag [18]. Nevertheless, research has also reported high levels of cross-linking in C-(N)-A-S-H [14]. Hydrocalumite (4CaO·Al_2_O_3_·13H_2_O, C_4_AH_13_) and katoite-type hydrogarnet phase (3CaO·Al_2_O_3_·6H_2_O, C_3_AH_6_) were identified in slag activated by sodium hydroxide, along with a high proportion of Al in octahedral sites, and a poorly crystallised C-(N)-A-S-H gel. Conversely, it was suggested that a high amount of Ca and Al was incorporated in the interlayer of the C-(N)-A-S-H phase in slag activated by sodium silicate [23]. Additionally, it has been demonstrated through quasi-elastic neutron scattering analysis that an initial C–(N)–A–S–H-type gel is exclusively created in slag activated with sodium silicate as a result of the precipitation of Ca and Al that dissolved from the slag and Si from the activator [24].

The stoichiometric ratio and structure of the C-(N)-A-S-H gel in AAS varies due to its versatility [4,5,14,18]. It is widely acknowledged that a cross-linked Al-substituted tobermorite (CSTM) model is suitable to describe the C-(N)-A-S-H gel formed in AAS [25,26,27,28,29]. According to this model, Al occupies bridging sites in the dreierkette structure without single bridging site vacancies. A highly cross-linked C-(N)-A-S-H gel is formed at an Al/Si ratio of 0.1 [30]. The Al content within C-(N)-A-S-H is crucial in determining the level of cross-linking. A highly cross-linked structure has a reduced capacity for Al intake, even though fivefold-coordinated Al may exist in the interlayer of the C-(N)-A-S-H structure [31]. A recent detailed analysis of the nanostructure of C-(N)-A-S-H gels reveals that a higher Ca content in the precursor promotes a C-(N)-A-S-H structure with low Al and high Ca levels. This structure displays a low degree of cross-linking. Furthermore, Al substitution is identified at multiple sites in the C-(N)-A-S-H structure, including cross-linking, bridging, and pairing sites [32]. 

Despite intensive research and significant progress in understanding the primary hydration products in AAS, there is limited knowledge about the effects of variation in slag chemistry and activation conditions on the hydration kinetics and hydrate assemblage. Studies on these aspects have mostly been parallel, with only a handful of works [3,4,5] that have evaluated both aspects. Therefore, there is a strong need for further research in this area. Three types of slag with different chemical compositions and six activators containing varied concentrations of alkali and silicate were chosen for analysis. The purpose of this study was to investigate the impact of variation in slag–activator on the early-stage hydration kinetics and hydrate assemblage. This was achieved using EDX analysis, alongside solid state ^29^Si and ^27^Al NMR techniques, to furnish data on the degree of hydration (DOH) and hydrate assemblage. Ultimately, these findings yielded an interaction model between slag and alkali silicate activator.

## 2. Materials and Methods

### 2.1. Materials

Three types of ground granulated blast furnace slags (referred to as S1, S2, and S3) were used in this investigation. S1, S2, and S3 were procured from Tengyan Minerals Manufacturing Co. Ltd. (Lingshou, Shijiazhuang, China), Shaoguan Iron & Steel Co., Ltd. (Shaoguan, China), and Wuhan Iron & Steel Group Metal Resources Co., Ltd. (Wuhan, China), respectively. S1, S2, and S3 have a d_50_ of 8.22, 8.41, and 11.54 μm, and a d_90_ of 24.13 μm, 31.26 μm, and 31.06 μm, respectively.

Table 1 presents the chemical compositions of the slags, which were analysed by XRF using UniQuant™ software (OXSAS 2.5). The glass content of the anhydrous slag was deduced from its XRD, recorded between 22–38° 2θ angles, according to the Chinese standard GB/T 18046-2017 Appendix C [33]. The mineral compositions of the slags are outlined in Table 2. It was suggested that the presence of calcite in S1 was due to the surface weathering of the slag during storage [34]. The percentage of calcite was 3.4 wt.%, as determined via thermogravimetric analysis.

Water glass solution (WG), with a molar modulus of 2 and a solid content of 44 wt.%, was purchased from Hengli Chemicals Co., Ltd. (Tongxiang, Jiaxing, China). Analytical reagent grade sodium hydroxide powder was purchased from Xilong Scientific (Shantou, China).

Deionized water was used in all experiments. All materials were obtained in their as-received state without additional purification.

### 2.2. Methods

#### 2.2.1. Sample Preparation

The AAS samples were prepared using a water-to-slag ratio of 0.45, which includes the water from the alkaline solutions. The alkaline solutions, namely, sodium silicate and sodium hydroxide, were prepared 24 h prior to use. The modulus of the sodium silicate solution was adjusted with sodium hydroxide solution. The dosages of the alkali activators were 3 wt.% and 5 wt.% Na_2_O (based on slag mass), respectively. Table 3 shows the different combinations of activators used. The slag powder was mixed with an alkaline solution in a 20 mL PE bottle using a vortex vibrator to prepare the samples. The prepared samples were then sealed and stored at 20 ± 2 °C. After 3 days of hydration, the samples were cut into small pieces and immersed in isopropanol to stop hydration. The samples were dried under a vacuum and stored in the desiccator. The specimens intended for energy dispersive X-ray (EDX) mapping analysis were impregnated with epoxy resin (EPO-25 from Mega Instruments Suzhou Co., Ltd. (Suzhou, China)) under a vacuum. After curing, the surface of the specimen was methodically polished using sandpapers from 400, 800, and 1200 mesh, along with ethanol as a lubricant. The polished samples were coated with gold by using a 108 Auto sputter coater from Cressington Scientific Instruments (Oxhey, UK). The samples for analysis by nuclear magnetic resonance (NMR) and X-ray diffraction (XRD) were manually ground in an agate mortar using a pestle.

#### 2.2.2. Characterization Methods

##### EDX

A Zeiss Gemini 300 field emission scanning electron microscope (FESEM) (Zeiss Group, Jena, Germany) fitted with an Oxford Instruments X-Max system (Abingdon, UK) was deployed for EDX analysis. The device functioned in the secondary electron mode. For point analysis, EDX information was gained at 15 kV to attain a good resolution, with peaks identified by K_α1_-lines. Roughly 15–20 points were examined at various spots for each specimen, with no points recorded in the flat region. For this study’s mapping analysis, a field of view measuring approximately 118 μm × 89 μm was scanned with 628 × 473 image resolution and 0.188 μm × 0.188 μm pixel size. Five to six images were collected per sample, as EDX mapping provides accurate analysis with relatively few images [35].

##### Degree of Hydration (DOH)

**Figure 1:**
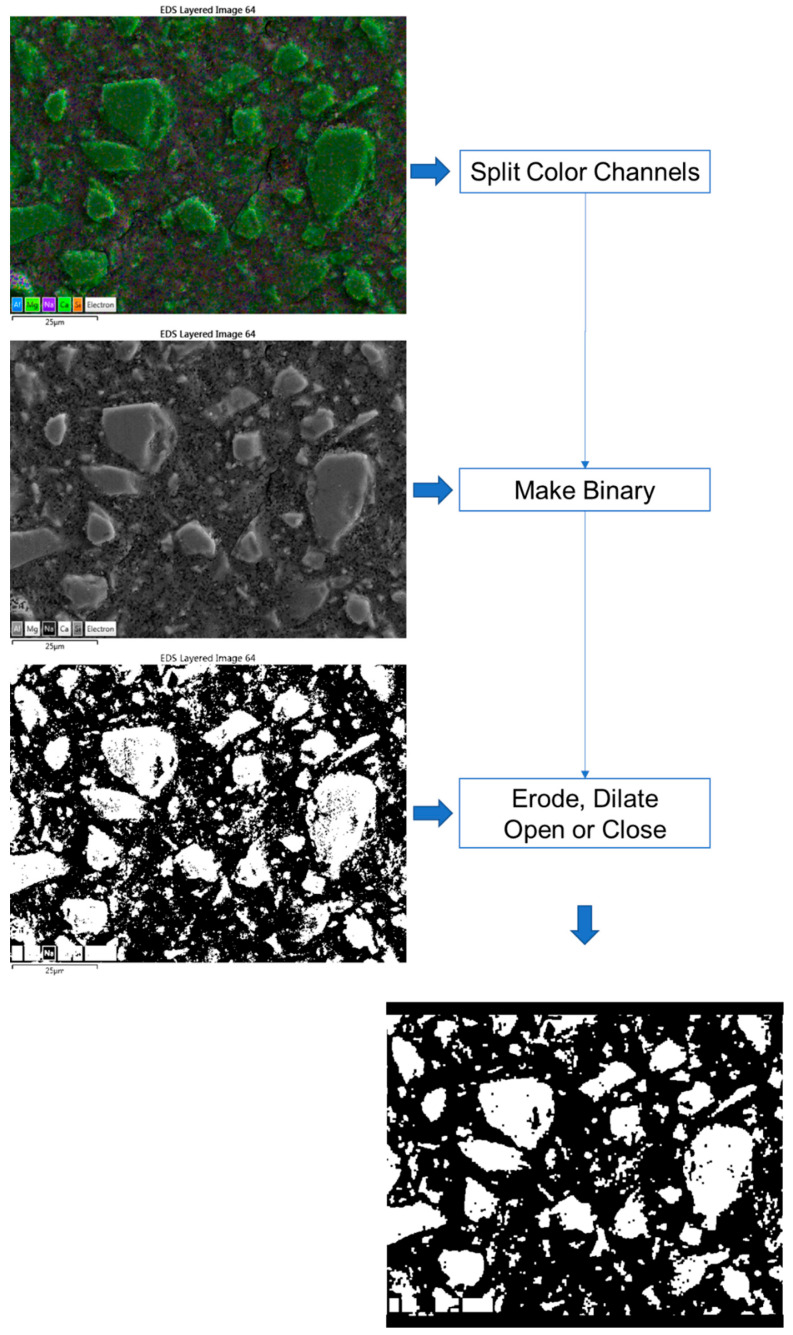
Image processing steps via ImageJ (the texts are the menu commands in the software).

Figure 1 depicts an EDX map image that is colour coded using five primary elements, namely, blue—Al, purple—Na; light green—Mg, green—Ca, and Orange—Si. The volume fraction of fly ash in the blended cement was successfully quantified by employing SEM-EDX mapping techniques [35,36]. Similarly, to determine the volume of the anhydrous slag, we first separated the RGB colour channels in the image. The resulting green channel image was then converted to a binary image, followed by erode, dilate, open or close instructions, either individually or collectively, similar to the process described in the literature [37,38]. All the treatments were performed using ImageJ software 1.54d. Si-rich particles were identified using the red channel image in certain cases. The DOH was calculated using the subsequent equation:

$$\mathrm{DOH}=100\;\times \;\left(1\;-\;\frac{V_{\mathrm{slag}}\left(t\right)}{V_{\mathrm{slag}}\left(t=0\right)}\right)$$

where V_slag_(t) is the volume fraction of the anhydrous slag at the time t and V_slag_(t = 0) is its volume calculated from the material mix design.

If there were no notable discrepancies between the processed and original images, particularly when it was difficult to distinguish the contrast between the anhydrous slag particles and the interstitial space, we employed a pretreatment by using the magnetic lasso tool in Photoshop. This technique was used to select and erase the slag particles present in the images.

##### NMR

Magic angle spinning nuclear magnetic resonance (MAS-NMR) spectra of ^29^Si and ^27^Al were obtained using a JEOL JNM-ECZ 600R spectrometer (JEOL, Tokyo, Japan) with a magnetic field strength of 14.1 T. The ^29^Si NMR measurements were taken at a spin rate of 5 kHz using an 8 mm HXMAS probe, with 90° pulses and a relaxation delay of 60 s in single-pulse mode. The spectra were externally referenced to Trimethylsilylpropanoic acid (TSPA) at 1.249 ppm. The measurements for ^27^Al-NMR were conducted using a 3.2 mm HXMAS probe, with a spin rate of 15 kHz. Single-pulse mode was employed, utilizing 90° pulses with a relaxation delay of 5 s. The ^27^Al-NMR spectra were externally referenced to AlK(SO_4_)_2_∙12H_2_O powder at −0.21 ppm.

It should be noted that in disordered solids, charges around the observed nucleus with a spin quantum number > 1/2 result in an electric field gradient (EFG) distribution. One example of this is found in the ^27^Al-NMR spectra of glasses [39]. The Czjzek model effectively simulates the ^27^Al NMR spectrum that is broadened due to quadrupolar effects in disordered solids, including slag [40]. Thus, in this study, the ^27^Al-NMR spectra underwent fitting using the Czjzek model via Dmfit software (release #20211004test) [41].

##### XRD

XRD analysis was carried out using a Bruker D8 Advance X-ray diffractometer instrument (Bruker, Billerica, MA, USA), operating at 40 kV and 40 mA. Cu-Kα radiation (λ = 1.5406 Å) was used in the θ-2θ configuration, with an angular range of 5–60° 2θ. The scan step was 0.0190° and the scan step time was 42.2 s.

## 3. Results and Discussion

### 3.1. Degree of Hydration

**Figure 2:**
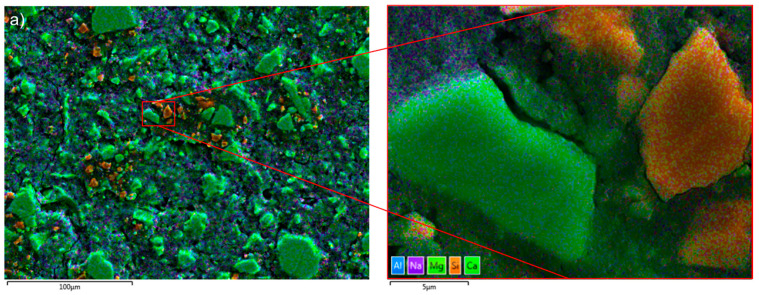

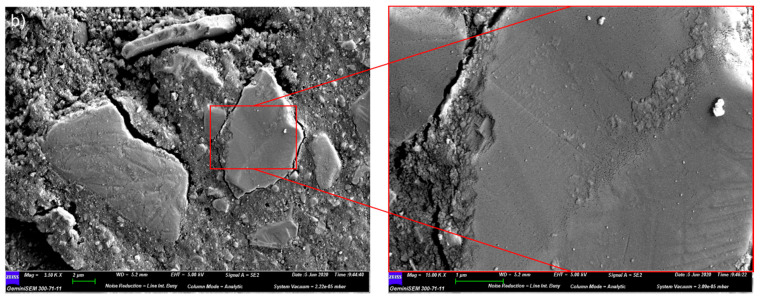
(**a**) The EDX mapping image of the Si-rich particles (in orange) in S2-A50 (false colours coded to the different elements: blue—Al; purple—Na; light green—Mg; green—Ca, and Orange—Si); (**b**) the SEM image of the Si-rich particles in sample S2-A50.

Enrichment of Ca and Al was found at the solid–liquid surface of slag-like glass which dissolved in an alkaline solution [42]. Figure 2a shows that the hydrated slag particles were abundant in Ca (green), while the Al-rich phase (blue) was dispersed in the hydration products, supporting the dissolution–reprecipitation mechanism. Furthermore, some Si-rich particles (orange) were noticed. Polishing marks were apparent on the particles’ surface (Figure 2b), implying a possible surface modification. In this scenario, it is possible that the polishing process generated Si-rich particles by removing Ca and Al from the surface. Nevertheless, the sharp contrast between the slag particles and the hydrates assists in the segregation of the particles from the AAS paste when determining the DOH of AAS.

The DOH of different combinations of slag–activator was determined using the method outlined in Section 2.2.2, as shown in Table 4. Generally, the DOH of the slag rises with its basicity (refer to Table 1) and the alkaline concentration, in the absence of silicate anions. The impact of the silicate modulus on the DOH of the three slags is varied when silicate anions are present. On the one hand, the DOH for S1 reduces when the modulus increases, whereas the trend is opposite for S3. On the other hand, S2 attains the highest DOH at a modulus of 1. Previous studies have documented that the DOH of slag activated by NaOH is more significant than that activated by water glass (M_s_ = 1) at the early stages [15,43]. The hinderance of slag dissolution by silicate anions in the starting solution can be comprehended due to the promotion of rapid formation of a poorly crystallised C-(N)-A-S-H gel via the presence of high concentrations of silicate in the vicinity of slag particles [44]. However, this explanation’s inconsistency with the results of S2 and S3 in the water glass-activated system should be noted. Nonetheless, the results suggest a strong dependence of the activator effect on the DOH.

### 3.2. Hydrate Assemblage

**Figure 3:**
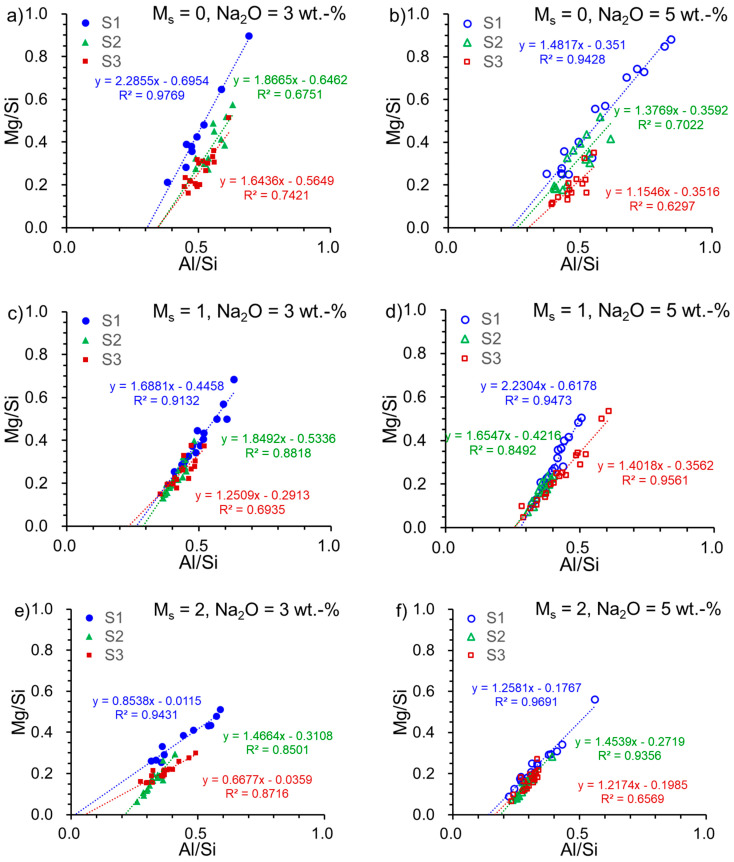
Mg/Si versus Al/Si in the alkali-activated slag pastes with various slag–activator combinations: (**a**) S1/S2/S3-A30; (**b**) S1/S2/S3-A50; (**c**) S1/S2/S3-A31; (**d**) S1/S2/S3-A51; (**e**) S1/S2/S3-A32; (**f**) S1/S2/S3-A52 (blue solid and open circles refer to the samples associated with S1; green solid and open triangles refer to the samples associated with S2; deep red solid and open squares refer to the samples associated with S3).

EDX analysis was conducted on the interstitial space of the pastes. The Mg/Si ratio and Al/Si ratio were deduced from the elements’ percentage and are depicted in Figure 3. A linear regression was carried out on Mg/Si-Al/Si plots to derive the slope and the intercept, indicating the Mg/Al ratio in hydrotalcite and Al/Si ratio in C-(N)-A-S-H, as described in the literature [4,45]. This method has been employed to determine the mean Al/Si ratio in C-A-S-H, and it correlated closely with the ratio acquired from NMR results [46]. Results for the slope are presented in Table 5. The Mg/Al ratio in the hydrotalcite phase is typically between 2–5 [47,48], with a few values surpassing 2. However, these low values reported here may not be directly related to the Mg/Al ratio in hydrotalcite. In reality, hydrotalcite and C-(N)-A-S-H are intermixed at the nanoscale in alkali-activated slag [49]. Therefore, the Mg/Al ratio determined by EDX represents the total hydrate ratio and not solely the ratio in hydrotalcite. The unusual values come from S1-A32 (Mg/Al = 0.85) and S3-A32 (Mg/Al = 0.67), which are comparable to those of S1 (Mg/Al = 0.81) and S3 (Mg/Al = 0.55), respectively. These readings hint that a small quantity of hydrotalcite was formed in these samples.

The intercept of the slope on the Al/Si axis was used to speculate Al/Si in the C-(N)-A-S-H gel [4]. It was reported that the Al/Si ratio of pure C-(N)-A-S-H was less than or equal to 0.20 when the molar Ca/(Si + Al) ratio fell within the range of 0.7–1.3 [50,51,52]. At an Al/Si ratio of 0.19, Al replaces a half-bridging tetrahedron in a fully polymerized C-S-H structure, while at a ratio greater than 0.19, some LDH phases such as calcium hemicarbonate hydrate were precipitated [51]. Strätlingite formation was observed at Al/Si ratios greater than 0.20, according to a previous study [50]. Based on our investigation of slag–activator combinations, the Al/Si ratio (intercept in Table 5) in the pastes, excluding S1-A32, S1-A52, S2-A52, S3-A32, and S3-A52, exceeded the limit (0.20). This indicates that more Al was incorporated into the hydrates other than C-(N)-A-S-H. A notably low value for sample S1-A32 also suggests that Al was not incorporated into the hydrates, which is in line with its low DOH. Conversely, sample S3-A32 exhibits a low Al/Si ratio but high DOH. It should be pointed out that the chemical composition of S1 and S3 is similar. Thus, the findings indicate that, apart from the chemical composition, the glassy characteristic of the slag has a crucial influence on AAS hydration.

**Figure 4:**
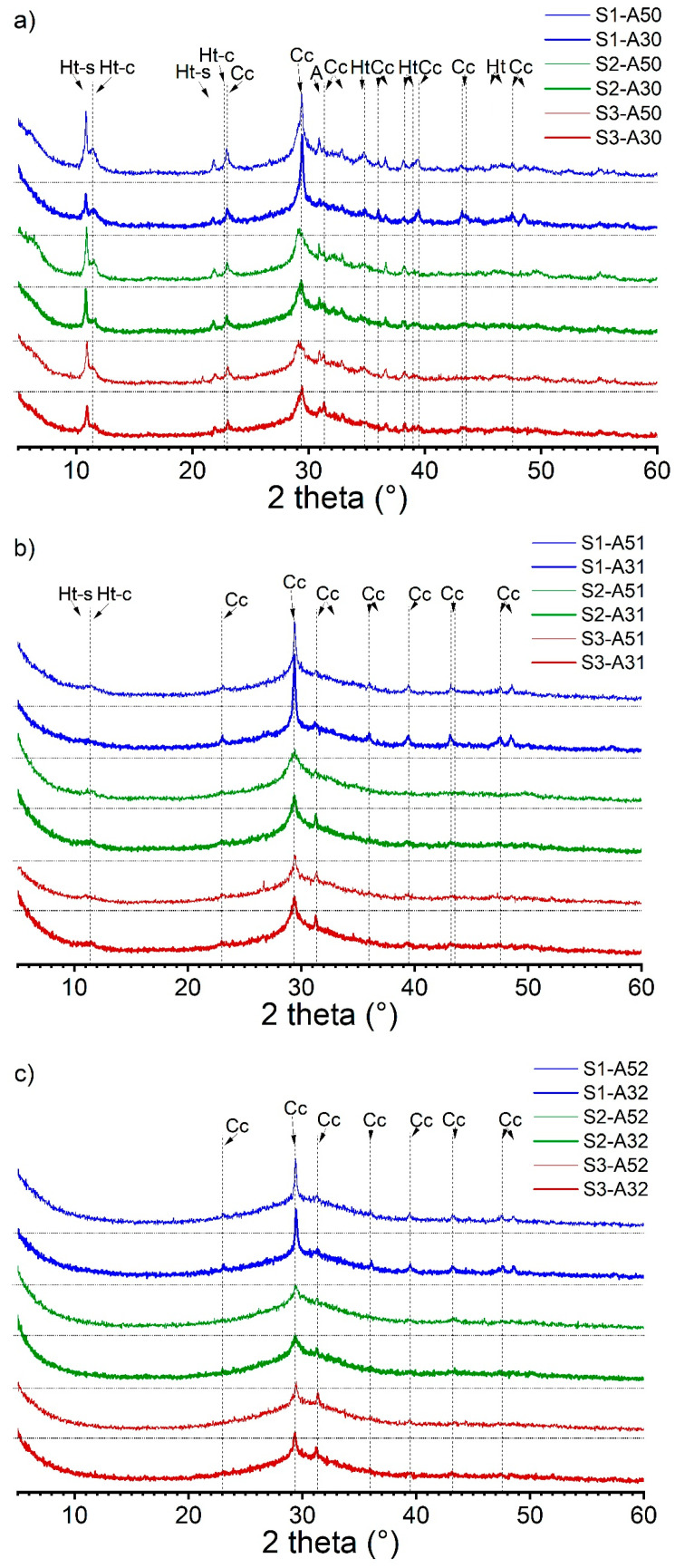
XRD spectra of the alkali-activated slag pastes with various slag–activator combinations: (**a**) S1/S2/S3-A30/A50; (**b**) S1/S2/S3-A31/A51; (**c**) S1/S2/S3-A32/A52 (Ht-s: Mg-Al LDH with polysulfide anions in the interlayer; Ht-c: Mg-Al LDH with carbonate anions in the interlayer; Cc: calcite; A: åkermanite).

To reinforce the EDX analysis of the phase assemblage, we conducted XRD analysis as illustrated in Figure 4. The XRD spectra were categorized based on the activator’s modulus. We observed that the crystalline phases in AAS pastes were affected by the activator’s modulus. As the modulus increased, the intensity of the hydrotalcite peaks decreased and eventually vanished. It should be noted that the disappearance of hydrotalcite peaks in the spectra does not necessarily indicate their absence in the hydrates. The C-(N)-A-S-H phase was combined with the XRD amorphous hydrotalcite phase, resulting in a low Mg/Al ratio (<2) in the pastes (refer to Table 5). Calcite was identified as the major crystalline phase, which formed due to the carbonation of the hydrates during sample preparation. Partial calcite content was also introduced by the anhydrous slag S1. The XRD spectra validated the presence of two types of hydrotalcite, also referred to as Mg-Al Layered Double Hydroxide (Mg-Al LDH), one of which contained carbonate in the interlayer due to carbonation of the hydrates. The occurrence of polysulfide in the interlayer was evidenced by its d-spacing of 0.817 nm, equivalent to a 2θ degree of 10.8, which was supported by earlier XRD findings in the literature [53,54].

**Figure 5:**
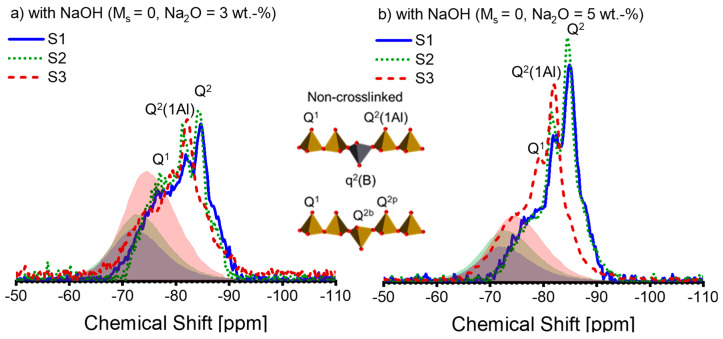

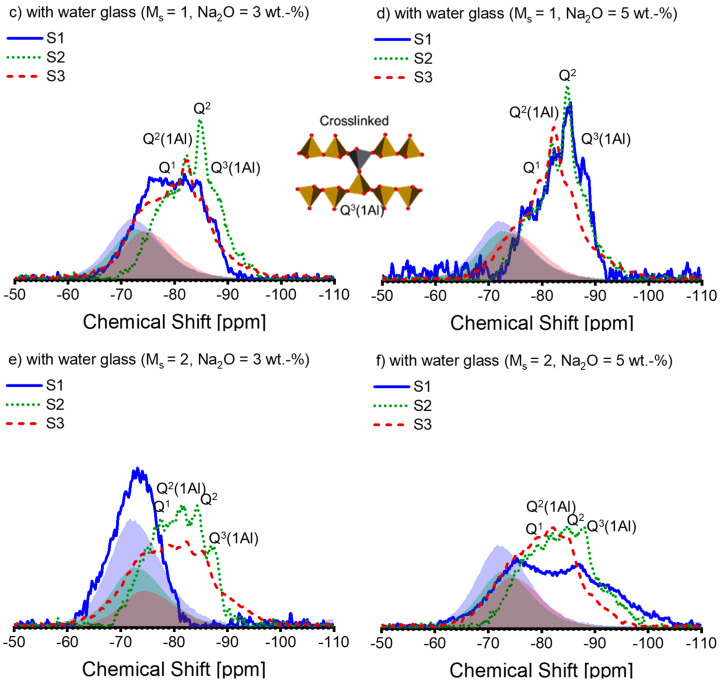
The ^29^Si MAS-NMR spectra of alkali-activated slag pastes with different alkali–activator combinations: (**a**) A30; (**b**) A50; (**c**) A31; (**d**) A51; (**e**) A32; (**f**) A52 (blue solid line: paste associated with S1; green dotted line: paste associated with S2; deep red dashed line: paste associated with S3; blue, green, and deep red shadows represent the spectra of the anhydrous slag S1, S2, and S3 scaled by the corresponding DOH listed in Table 4, respectively). Note: the intensity of the spectra is normalized.

As the XRD analysis was unable to determine the crystallite in the AAS pastes, the NMR analysis was conducted. The ^29^Si NMR spectra of the AAS pastes are depicted in Figure 5. The anhydrous slags’ spectra scaled by DOH are plotted for comparison purposes, shown in the shadows. It can be observed that the anhydrous slags’ spectra are not entirely superimposed on those of the AAS pastes. Therefore, subtracting the spectral line of the anhydrous slags, as described in reference [43], to extract the hydrate assemblage is not a suitable method. Consequently, peak deconvolution is not carried out on the ^29^Si NMR spectra.

The activation conditions noticeably affect the ^29^Si spectral lines of the pastes (see Figure 5). As the alkali and silicate concentration increases, the spectral lines shift to more negative values, implying a polymerized silicate structure within the pastes [55]. According to [25,32,50,52], we attribute the peaks or shoulders at −78 ppm, −82 ppm, −85 ppm, and −87 ppm to Q^1^, Q^2^(1Al), Q^2^, and Q^3^(1Al), respectively. A slight differentiation can be observed between the spectral lines of S1-A30 and S2-A30 (see Figure 5a), which is repeated in between those of S1-A50 and S2-A50 (see Figure 5b). As a result, the spectral lines of S3-A30 and S3-A50 suggest that Q^2^(1Al) dominates over Q^2^ within the C-(N)-A-S-H. Figure 5c,d reveal that for the activators A31 and A51, the associated pastes of S1 and S2 display a more cross-linked C-(N)-A-S-H structure, which is indicated by the raised Q^3^(1Al) peak. This signifies a low Al/Si ratio in C-(N)-A-S-H [31]. The formation of the polymerized C-(N)-A-S-H structure is impeded in S1-A31 due to the presence of silicate anions, alongside low alkali concentration. On the contrary, S3-A31 shows a higher amount of Q^2^(1Al), demonstrating a higher Al/Si ratio in C-(N)-A-S-H. Upon activation by A32 (Figure 5e) and A52 (Figure 5f), the paste associated with S2 displays a higher proportion of Q^3^(1Al) peaks, while negligible structural alterations are observed in the paste of S1-A32. With higher levels of alkali concentration, the paste of S1-A52 demonstrates structural development. The results indicate that silicate anions present in low alkali concentration slow down the reaction for specific slags, which has previously been reported in [16]. Q^2^(1Al) and Q^2^ continue to dominate in the pastes related to S3. It is possible that a significant amount of Si and Al released from S3 was incorporated into the C-(N)-A-S-H structure, as a high DOH of S3 was observed. Additionally, the spectral lines fall within the −90 ppm to −100 ppm region when the silicate activators are present. Previous reports have assigned Q^4^(3Al) (centre at -93 ppm) or Q^4^(4Al) (centre at −89 ppm) to a disordered nano-sized zeolite phase in AAS [25]. It has been reported that a zeolite phase is formed in AAS in instances where there is inadequate Mg in the system [56]. The starting solution contains an adequate amount of silicate anions, but the paste reveals insufficient Mg as shown by the Mg/Al ratio; hence, it is plausible that an intermediate zeolite-like product was formed in these pastes.

**Figure 6:**
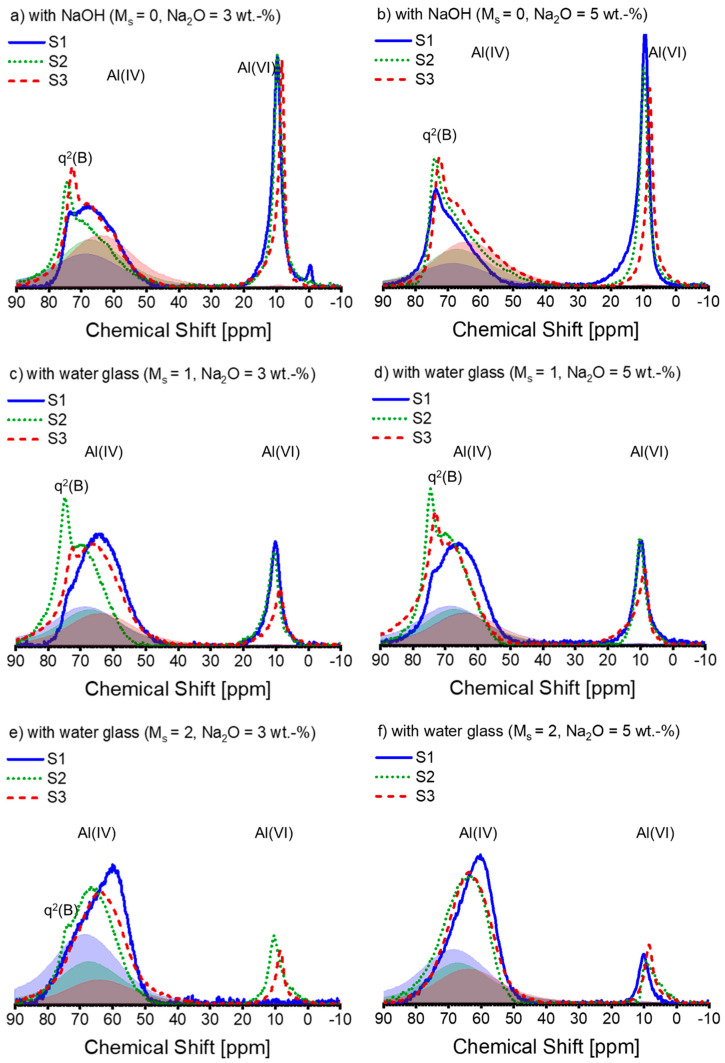
The ^27^Al MAS-NMR spectra of alkali-activated slag pastes with different alkali–activator combinations: (**a**) A30; (**b**) A50; (**c**) A31; (**d**) A51; (**e**) A32; (**f**) A52 (blue solid line: paste associated with S1; green dotted line: paste associated with S2; deep red dashed line: paste associated with S3; blue, green, and deep red shadows are the spectra of the anhydrous slag S1, S2, and S3 scaled by the corresponding DOH listed in Table 4, respectively). Note: the intensity of the spectra is normalized.

The ^27^Al NMR spectra for the AAS pastes are displayed in Figure 6. The profile of the spectral lines is determined by the combination of slag chemistry and silicate module in the alkaline solution, which is distinct from that observed in the ^29^Si NMR spectra. Due to the possibility of inconsistent DOH in Al and slag, peak deconvolution is not performed on the entire ^27^Al NMR spectra.

The literature suggests that the protruded narrow peak found at 72–74 ppm within the ^27^Al NMR spectra identifies Al(IV) incorporated into non-cross-linking bridging tetrahedra connected to the Q^2^(1Al) site (termed q^2^(B), akin to Q^2^(1Al) in the bridging position of the dreierkette structure) in the C-(N)-A-S-H gel [57,58]. This Al(IV) resonance is identified as tetrahedral Al on the bridging location in the C-A-S-H structure, as per Sun et al. [50]. Nonetheless, in contrast to S1, significant Al incorporation in C-(N)-A-S-H is observed in most of the pastes linked with S2 and S3.

**Figure 7:**
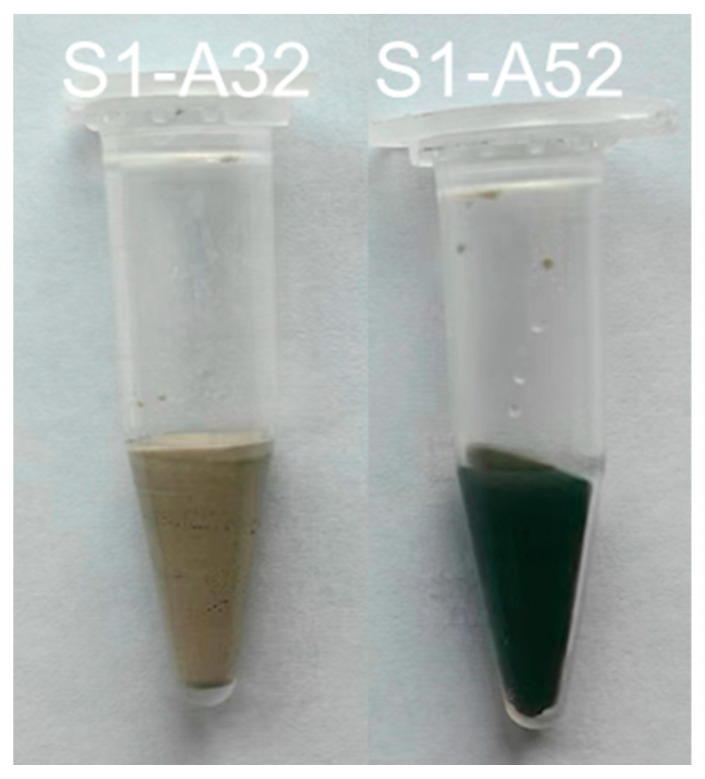
Pastes S1-A32 and S1-A52 after 3 days of hydration.

As the concentration of silicate in the initial solution increases, the proportion of Al(VI) decreases. Under each activation condition, the area of the Al(VI) peak associated with S2 is almost equal to or slightly smaller than that of S1, while the peak size of S3 is always smaller than that of S1 and S2, except for S1-A32. It is not unexpected that no Al(VI) peak occurred in the ^27^Al spectrum of S1-A32 (Figure 6e). Indirect evidence emerged during the sample preparation as the paste of S1-A32 retained a yellowish-white hue instead of turning blue or green during the experimental period. The transient development of a blue to dark green colour developed during slag hydration occurs due to the presence of polysulfide anions in the interlayer of the LDH structure under anoxic conditions, as reported in [59,60]. Additionally, [53] corroborates these observations, as Mg-Al LDH containing polysulfide anions also exhibited a dark greenish hue. Since the green colour was not present in the paste of S1-A32 (Figure 7), the visual observation agrees with the ^27^Al spectrum of S1-A32.

**Figure 8:**
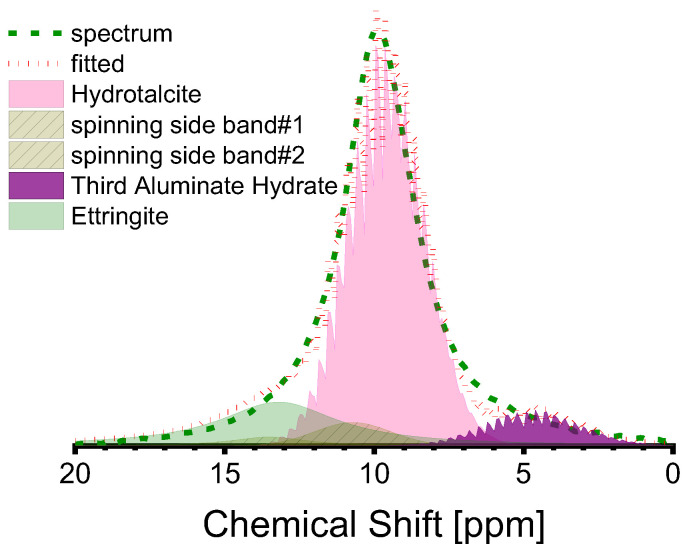
Deconvolution of Al(VI) peak in the ^27^Al MAS-NMR spectra of S2-A50 (red dotted line is the cumulative spectrum, which is composed of the sub-peaks as shown in pink, purple, and olive shadows).

The peak at 10 ppm in the Al(VI) spectra is complex, with several sub-peaks being identifiable through peak fitting, as illustrated in Figure 8. The sub-peak at approximately 10 ppm is attributed to the Al(VI) of hydrotalcite [26,33,44], while the sub-peak at about 13 ppm is attributed to ettringite, based on existing research [40,61]. Ettringite is not a commonly reported component in the hydrate assemblage of AAS, although its stability has been predicted when the mass fraction of SO_3_ in the anhydrous slag is equivalent to 2% [62], as is the case in this study. The sub-peaks around 5 ppm should be attributed to the third aluminate hydrate (TAH), which can exist as a disordered aluminate hydroxide or a calcium aluminate hydrate, as a separate phase, or as a precipitate on the surface of the C-A-S-H gel [10,25,32,55]. The minuscule sub-peak located approximately at 0 ppm was recently identified as [AlO_2_(OH)_4-x_(H_2_O)_x_]^(5−x)^ (x = 1,2) (see Figure 6a). According to a previous study [11], the Al(VI) in C-(N)-A-S-H relates to [AlO_2_(OH)_4−x_(H_2_O)_x_]^(x−5)^ (x = 0, 1, 2) rather than TAH. Thus, the Al(VI) chemical shift originating from the C-(N)-A-S-H gel is contingent upon the coordination number of OH^−^ and H_2_O. Table 6 presents the fitting outcomes. Based on the findings, the dominant hydrate assemblage of Al(VI) is hydrotalcite, which comprises over 50% of the assemblage, accompanied by ettringite and TAH (or [AlO_2_(OH)_4−x_(H_2_O)_x_]^(x−5)^ species in reality).

### 3.3. Discussions

Based on the previous findings, the significance of the Al hydrate assemblage in the interplay of slag and alkali activators is emphasized. The rate of dissolution of Al in slag-like glass when subjected to alkaline solution was observed to have a correlation with the ratio of non-bridging oxygen atoms (NBO) to oxygen atoms in tetragonal coordination (NBO/T), which is a classical index for the degree of polymerisation in glass science [63]. This term was coined to indicate the level of depolymerization degree in the amorphous phase of fly ash, which strongly correlated with the amount of heat generated during hydration after 7 days in a 50:50 blend of cement and fly ash [64]. Correspondingly, the dissolution of Si and Al species from the glassy structure automatically gives rise to NBO in the solution, regardless of the rapid formation of hydration products like the C-(N)-A-S-H gel. Based on this analogy, we presented a suggested pseudo NBO/T index—NBO/T*—to reflect the depolymerization of the glass structure resulting from exposure to alkaline (silicate) solutions:

$$\mathrm{NBO}/T^{*}=\frac{2\;\times \;\left({\;X}_{\mathrm{Ca}}{+X}_{\mathrm{Mg}}\;\right){+X}_{K\;}{+X}_{{\mathrm{Na}}^{*}}{\;-\;X}_{\mathrm{Al}}}{X_{{\mathrm{Si}}^{*}}{+X}_{\mathrm{Al}}}$$

where X_Ca_, X_Mg_, X_K_, and X_Al_ are the molar fractions of calcium, magnesium, potassium, and aluminum in the slag, respectively, while X_Na*_ and X_Si*_ are the molar fractions of sodium and silicon in the slag and activator, respectively. 

**Figure 9:**
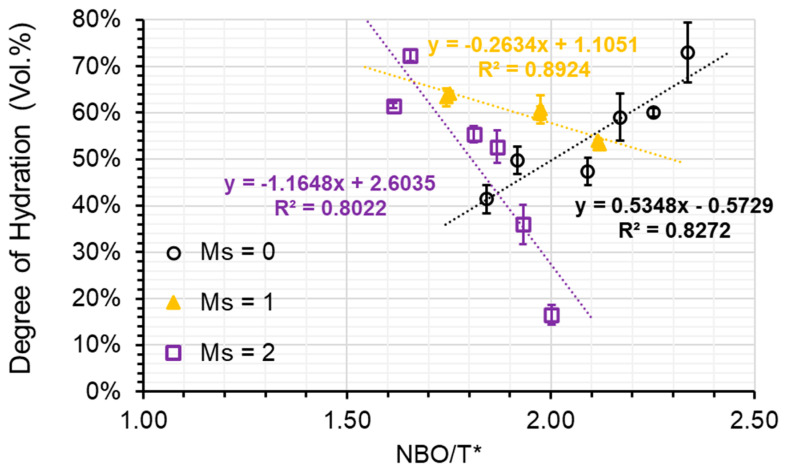
The DOH plotted against the NBO/T* ratio (black open circle refers to the samples associated with A30 and A50; yellow triangle refers to the samples associated with A31 and A51; violet open square refers to the samples associated with A32 and A52).

Plotting NBO/T^*^ against DOH reveals that a relatively linear relationship can be found for the AAS pastes grouped by the activator’s modulus (refer to Figure 9). With increasing modulus, the slope of the linear regression decreases from positive to negative in a clockwise direction. When a low modulus activator (M_s_ = 0) was utilised, the degree of hydration (DOH) within the paste of S1-A50 was discovered to be 72.9%, while in the paste of S3-A30, it was merely 41.4%. Nevertheless, when a high modulus activator (M_s_ = 2) was employed, the DOH in the paste of S1-A32 plummeted to only 16.5%, whereas in the paste of S3-A32, it soared to a remarkable 72.4%. The differences in DOH observed can be credited to distinct levels of depolymerization of the slags. Meanwhile, the results imply that the depolymerization increment causes unfavourable impacts on slag hydration when high silicate concentration is present in the activator. To decrease the difference of DOH in pastes, a moderate modulus activator (M_s_ = 1) should be employed, resulting in less than 10% variances among the AAS pastes. This finding suggests that a balance was nearly reached between the advantageous impact of slag depolymerization degree and the detrimental effect of the alkali silicate activator on slag hydration.

**Figure 10:**
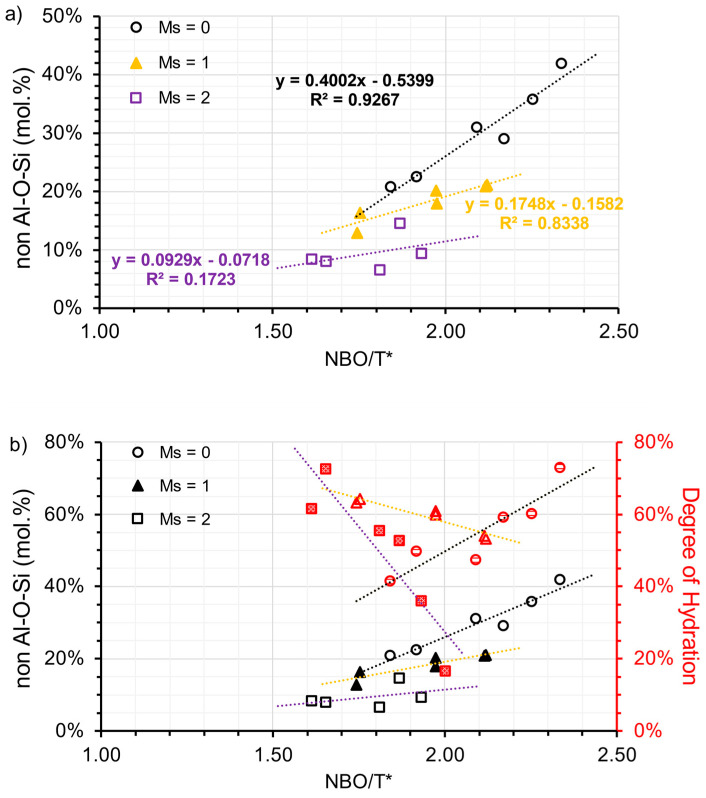
(**a**) the ratio of Al(VI) from LDH (designated as non-Al-O-Si) and (**b**) the relationship between the ratio of non-Al-O-Si and DOH, plotted against the NBO/T^*^ ratio and grouped by the modulus of the activators (open circle refers to the samples associated with A30 and A50; solid triangle refers to the samples associated with A31 and A51; open square refers to the samples associated with A32 and A52).

To approximately determine the hydrate assemblages, the peak area within the range of 40–90 ppm [Al(IV)] and −10–20 ppm [Al(VI)] were integrated, respectively. It is justifiable to use peak area to denote the proportion of different Al states, since the secondary order quadrupolar broadening effect on the line shape of ^27^Al nuclei is consistent in all phases [61]. The percentage of Al(VI) in the total Al phase (including the anhydrous phase) can be calculated and shown in Table 7. To distinguish Al in different hydrate assemblages, it is necessary to exclude Al(VI) in C-(N)-A-S-H. Subsequently, by subtracting the ratio of total TAH or de facto [AlO_2_(OH)_4−x_(H_2_O)_x_]^(x−5)^ (as shown in Table 7), a new Al(VI) ratio termed the non-Al-O-Si ratio is plotted against NBO/T*. A clearer linear relationship between NBO/T* and the Al hydrate assemblage is then presented in Figure 10a. As the modulus increases, the slope of the linear regression also decreases in a clockwise direction, similar to the relationship between NBO/T* and DOH. The activation condition plays a crucial role in controlling the formation of the Al(VI) hydrates. In addition, NBO/T* proves effective in interpreting the results for samples using a low modulus activator. Hydrotalcite formation is reportedly governed by the free available Mg dissolved during the alkaline activation process [45], which is inadequate during the early stages. There is indisputable evidence that the dissolved Al is incorporated into the C-(N)-A-S-H gel, particularly when the alkali silicate activator is present. The dissolved Al also precipitates in the form of other LDH phases including calcium monocarbonate hydrate, depending on the amount of polysulfide replaced by carbonate.

If the hydration degree of Al aligned with the DOH in AAS, the proportion of hydrotalcite found in the hydrate can be estimated by dividing the non-Al-O-Si ratio by DOH. Using a low modulus activator (M_s_ = 0), the proportion of hydrotalcite in the hydrate varied between 49% and 66%. When a medium modulus activator (M_s_ = 1) was used, this proportion dropped to a range of 20% to 40%. Finally, when a high modulus activator (M_s_ = 2) was used, the proportion reduced to a range of 11% to 27%. The results suggest that the increased DOH levels of S1-A30/A50 are enhanced due to the formation of hydrotalcite, whereas the elevated DOH of S3-A32/A52 are associated with the formation of C-(N)-A-S-H, evident from the non-Al-O-Si ratio and DOH correlation (refer to Figure 10b).

**Figure 11:**
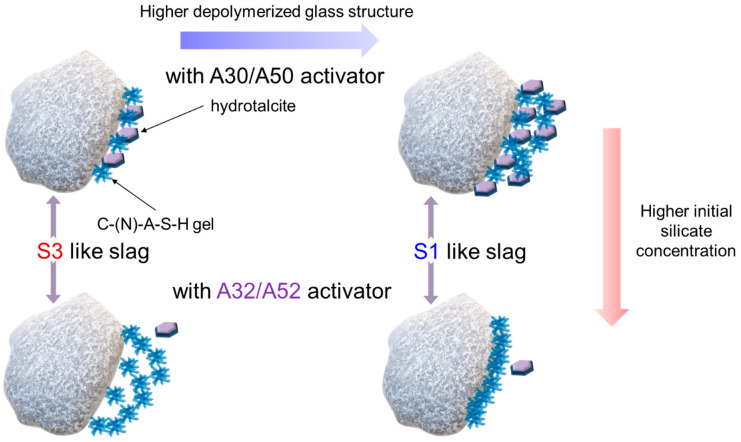
Schematic illustration of interaction between slag and alkali silicate activator.

The early-stage dissolution kinetics of slag-like calcium aluminosilicate (CAS) glass under alkaline conditions was elucidated using a dissolution–reprecipitation mechanism [42]. The mechanism proposes that a new phase is precipitated, which advances the reaction front at the interface between the CAS glass and the activating fluid. Precipitation of the new phase plays a significant role in the dissolution of the slag. By using the dissolution–reprecipitation mechanism and the outcomes of this study, the initial interaction between the slag and alkali silicate activator is proposed (Figure 11). If the slag has a more polymerized structure, the formation of both the C-(N)-A-S-H gel and hydrotalcite facilitate slag hydration. However, in the case of a highly depolymerized structure of the slag, the formation of hydrotalcite plays a significant role in achieving the high DOH of the slag. As the concentration of silicate increases, the slag with a more polymerized structure continuously hydrates with the silicate anions in the solution, resulting in the precipitation of the C-(N)-A-S-H gel and small amounts of hydrotalcite. The slag with a highly depolymerized structure hydrates to form the C-(N)-A-S-H gel near the slag particles. The formation of a dense gel structure strongly hinders hydration, leading to a very low DOH.

## 4. Conclusions

This study affirms that the interaction between slag and alkali silicate activator follows the dissolution–reprecipitation mechanism, underscoring the significance of both the slag and activator composition. Consequently, a pseudo NBO/T index, termed NBO/T* index, was suggested by taking both the slag and activator composition into consideration during the dissolution of slag.

The research discovered a roughly linear correlation between the DOH of the slags and the NBO/T* index when the results were classified according to the activators’ modulus. As the modulus increased, the hydration of the slag with a highly depolymerized structure was retarded. In the most extreme cases, the hydration was significantly slowed down. Conversely, a high modulus promoted the hydration of the highly polymerized slag. The Al distribution in the hydrate assemblage illustrated the connection between the DOH and the hydration products. With an increasing modulus, the driving force of hydration was shifted from the hydrotalcite to the C-(N)-A-S-H gel. These findings present a new approach to AAS design by employing both the NBO/T* index and the activators’ modulus.

It is advisable to use a medium modulus activator (around 1) in AAS design, not only for its strength benefits but also for its ability to reduce the impact of slag compositional variations through the introduction of a relatively stable DOH and hydrate assemblage under this condition.

## Figures and Tables

**Table 1:** The chemical composition of the investigated slags (wt.%).

	SiO_2_	Al_2_O_3_	TiO_2_	MnO	CaO	MgO	Na_2_O	K_2_O	SO_3_ ^†^	Al/Si	Basicity ^‡^
S1	28.4	15.4	1.66	0.31	41.0 *	9.93	0.30	0.40	2.65	0.64	1.15
S2	31.9	14.5	0.71	0.21	42.8	6.71	0.42	0.49	2.23	0.53	1.07
S3	31.8	16.6	1.05	0.40	39.7	7.22	0.65	0.45	1.40	0.61	0.97

* The amount of calcium oxide from calcite was deducted; ^†^ SO_3_ contains both SO_4_^2−^ and S^2−^; ^‡^—weight fraction ratio of the oxides (CaO + MgO)/(Al_2_O_3_ + SiO_2_).

**Table 2:** The mineral composition of the investigated slags (wt.%).

	Amorphous	Åkermanite	Calcite *
S1	93.6	3.0	3.4
S2	96.4	3.6	not detected
S3	93.2	6.8	not detected

* The amount of calcite was determined by thermogravimetric analysis.

**Table 3:** Different alkali activator combinations (wt.%).

Item\No.	A30	A50	A31	A51	A32	A52
Na_2_O dosage	3%	5%	3%	5%	3%	5%
Modulus of water glass * (Na_2_O·nSiO_2_)	0	0	1	1	2	2
Molality of alkali concentration (mol/kg solvent) ^†^	2.15	3.58	2.15	3.58	2.15	3.58
Solid content ^†^ % by mass of solution	7.9%	12.6%	12.5%	19.3%	16.4%	24.6%

* Sodium hydroxide corresponds to a modulus of 0; ^†^—by counting additional water in the mix design.

**Table 4:** The DOH of the AAS samples (vol.%).

Slag\Activator	A30	A50	A31	A51	A32	A52
S1	60.1 ± 1.0	72.9 ± 6.5	53.2 ± 1.0	54.0 ± 0.2	16.5 ± 2.1	36.0 ± 4.2
S2	47.3 ± 3.0	59.1 ± 5.1	59.9 ± 1.5	60.8 ± 3.0	52.7 ± 3.5	55.4 ± 1.7
S3	41.4 ± 3.0	49.8 ± 2.9	63.3 ± 1.9	64.2 ± 0.6	72.4 ± 1.2	61.5 ± 0.6

Note: Standard error $\mathrm{SE}=\frac{\mathrm{SD}}{\sqrt{n}}$ was used.

**Table 5:** Mg/Al slope and intercept at Al/Si axis in Figure 3.

	Slag\Activator	A30	A50	A31	A51	A32	A52
Slope	S1	2.29	1.48	1.69	2.23	0.85	1.26
	S2	1.87	1.38	1.85	1.65	1.47	1.45
	S3	1.64	1.15	1.25	1.40	0.67	1.22
Intercept	S1	0.30	0.24	0.26	0.28	0.01	0.14
	S2	0.35	0.26	0.29	0.26	0.21	0.19
	S3	0.34	0.31	0.23	0.25	0.05	0.16

**Table 6:** The fitting outcomes of the Al(VI) peaks in the ^27^Al MAS-NMR spectra.

Slag–Activator	Relative Integral Area * (%)	Isotropic Chemical Shift (ppm)	Quadrupolar Coupling Constant (MHz)	Assignment
S1-A30	31.3	13.2 ^†^	/^‡^	ettringite
	56.0	10.0	1.18	hydrotalcite
	6.1	5.0	1.07	TAH
	3.1	0.0	1.15	TAH
S2-A30	80.5	10.2	1.85	hydrotalcite
S3-A30	24.0	13.2	/	ettringite
	44.5	9.1	1.38	hydrotalcite
	1.4	4.9	0.88	TAH
S1-A31	53.7	13.2	/	ettringite
	43.2	10.1	1.24	hydrotalcite
	3.1	4.9	0.87	TAH
S2-A31	63.9	13.2	/	ettringite
	33.9	10.1	1.04	hydrotalcite
	2.2	5.1	0.86	TAH
S3-A31	36.3	13.2	/	ettringite
	59.6	9.2	1.37	hydrotalcite
	4.1	4.2	1.21	TAH
S1-A32	N.A.	N.A.	N.A.	N.A.
S2-A32	27.9	13.2	/	ettringite
	59.6	10.2	1.42	hydrotalcite
	12.5	5.3	1.89	TAH
S3-A32	20.7	13.2	/	ettringite
	56.4	9.2	1.48	hydrotalcite
	22.9	5.4	1.17	TAH
S1-A50	36.7	13.2	/	ettringite
	50.2	9.4	1.14	hydrotalcite
S2-A50	17.4	13.2	/	ettringite
	63.1	10.0	1.21	hydrotalcite
	6.6	5.0	1.04	TAH
S3-A50	14.6	13.2	/	ettringite
	65.6	9.0	1.75	hydrotalcite
	19.8	5.2	1.15	TAH
S1-A51	21.7	13.2	/	ettringite
	66.2	10.1	1.31	hydrotalcite
	12.2	5.8	1.23	TAH
S2-A51	21.8	13.2	/	ettringite
	70.8	10.1	1.11	hydrotalcite
	7.4	5.0	0.97	TAH
S3-A51	25.8	13.2	/	ettringite
	57.0	9.1	1.17	hydrotalcite
	17.2	5.1	1.25	TAH
S1-A52	100	10.3	1.22	hydrotalcite
S2-A52	58.2	9.5	1.29	hydrotalcite
	41.8	5.0	1.17	TAH
S3-A52	70.8	9.1	1.23	hydrotalcite
	29.2	5.1	1.15	TAH

* Automatically calculated by Dmfit software, including the contribution of spinning side band; ^†^—isotropic chemical shift of ettringite was fixed at 13.2 ppm, which is adopted from ref. [40]; ^‡^—the line shape of the ^27^Al NMR spectrum of ettringite is Lorentzian, since the highly symmetrical coordination of Al in the crystal lattice experiences no quadrupolar effect [40].

**Table 7:** Ratios of Al(VI) and Al(VI) excluding that in C-(N)-A-S-H (designated as non-Al-O-Si) in total Al (%).

Ratio	Slag/Activator	A30	A31	A32	A50	A51	A52
Al(VI)	S1	41.0 *	21.8	2.5	48.1 *	23.7	9.3
	S2	38.5 *	20.6	16.6	36.1 *	19.4	11.1
	S3	30.4 *	13.5	10.3	28.0 *	19.7	11.8
non-Al-O-Si	S1	36.7	21.1	/	44.6	20.8	9.3
	S2	31.4	20.2	14.5	31.4	17.9	6.5
	S3	20.8	12.9	8.0	22.4	16.3	8.3

* Data were corrected by subtracting the contribution of the spinning side band; for the remaining samples, no significant spinning side band was observed in the spectra.

## Data Availability

The data presented in this study are available on request from the corresponding author.
